# Management of borderline ovarian tumours: a comprehensive review of the literature

**DOI:** 10.3332/ecancer.2014.403

**Published:** 2014-02-17

**Authors:** Alejandra Abascal-Saiz, Laura Sotillo-Mallo, Javier de Santiago, Ignacio Zapardiel

**Affiliations:** Gynaecological Oncology Unit, La Paz University Hospital, Madrid, Spain

**Keywords:** borderline ovarian tumour, management, fertility-sparing surgery

## Abstract

Borderline ovarian tumours differ from epithelial ovarian cancer by their low incidence, frequent association with infertility, low association with mutations in BCRA genes, different percentages of the most common histological types, early stage diagnosis, and high survival rate, even when associated with peritoneal involvement. They occur in younger women, which is why one of the objectives in these patients will be the preservation of fertility. The management of these tumours has been widely discussed and still continues to be controversial. The latest findings underscore the importance of full staging in both radical and conservative surgery, to choose the most comprehensive treatment and obtain an accurate prognosis. One of the objectives of this article shall be the in-depth review of the indications, benefits, and disadvantages of each type of surgery, as well as the usefulness of the medical treatment. In addition, the article aims to review follow-up guidelines and to clarify the main prognostic factors that affect recurrence and survival of these patients.

## Introduction

**Figure 1.**
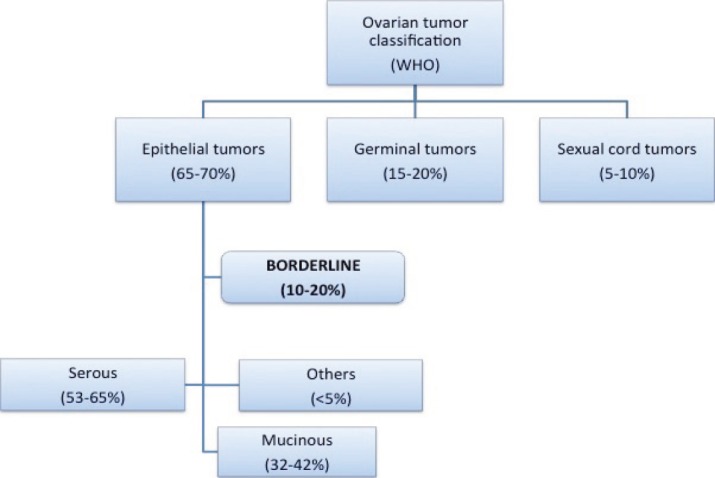
Ovarian tumour classification (WHO).

Borderline ovarian tumours (BOTs) have been qualified as low malignant potential tumours by the FIGO since 1971 [1–4]. They are classified within malignant epithelial ovarian tumours, constituting 10–20% of these (Figure 1) [2, 5].

Their incidence is low, and is calculated in European series at around 4.8/100,000 new cases per year [1, 5] and even lower in American series, between 1.5 and 2.5/100,000 cases per year [2, 6].

They occur in women at approximately 40 years of age (in 27–36% of cases the tumours occur at a younger age) [1, 6, 7], compared with an average occurrence at 60 years in the case of invasive carcinoma [1].

The risk and protective factors for the occurrence of BOT are similar to those of carcinoma; however, the association with mutations in *BCRA *genes is exceptional. In some studies, an increase in the incidence (two to four times greater) of serous BOT in women undergoing assisted reproduction techniques has been observed. This seems to have some correlation with the hormonal levels achieved during ovarian stimulation and the damage caused by repeated gonadal punctures [1, 3].

Some patients with BOT (16–30%) are asymptomatic when diagnosed and the discovery is incidental; nevertheless, when there are symptoms these are often non-specific, similar to other adnexal tumours, such as pelvic pain or abdominal distension [6, 8].

## Classification

Depending on their size, BOTs are classified according to the FIGO classification used for other ovarian tumours [9]; however, the majority of these tumours (70–80%) are diagnosed at stage I, compared with 25% of carcinomas [3]. A diagnosis of BOT in stages II and III is rare, and exceptional in stage IV [1, 7, 10].

Most of the BOTs, like carcinomas, are serous tumours, accounting for about 53–65%. Mucinous BOT constitutes between 32% and 42% of the total (compared with less than 10% of mucinous ovarian carcinomas). The rest of the BOTs (less than 5%) are composed of endometrial tumours, clear-cell tumours, Brenner’s tumours, and other unique histologies [1–3, 6].

### 1. Serous BOT

Tumours are bilateral in one-third of cases. These are associated with peritoneal implants in 35% of cases, of which up to 15–25% can be invasive implants, the omentum being the most common area affected [1–3, 6]. In addition, in advanced stages, these may be associated with lymphatic involvement in about 27% of cases, including the following in descending order of frequency: pelvic, omental and mesenteric, and paraaortic and supradiaphragmatic regions [6, 7].

Serous BOT can be further divided into two subtypes:

– Typical pattern (90%) is often a unilocular cystic mass with fine septa in its interior.
– Micropapillary pattern (10%) presents specific histological features (micropapillary appearance contiguous over > 5mm or in more than 10% of the tumour) [3, 11]. The latter has a worse prognosis since the majority are associated with a higher rate of recurrence in invasive form, a greater percentage of bilaterality and presence of invasive implants, and upstaging when performing restaging surgery [1, 2, 7, 10]. However, the latest publications suggest that serous BOT with micropapillary pattern and without implants (stage I) or with non-invasive implants (II and III) could have the same prognosis as serous BOT without micropapillary pattern. Therefore, malignancy is more closely related to the presence and invasiveness of implants [2].

### 2. Mucinous BOT

These tend to be larger than serous BOT and have either a unilocular or multilocular cystic structure, with fine septa in their interior and intramural nodules [3]. Peritoneal implants are very uncommon (15%), and when they occur, a mixed histology as well as the presence of pseudomyxoma peritonei must be ruled out. These are considered a differentiated entity, in which peritoneal involvement of a mucinous carcinoma is primarily of digestive origin, generally of the appendix [1, 2].

They are divided into two subtypes:

– Intestinal (85–90%): the majority of these are unilateral and in the case of a bilateral occurrence, primary intestinal cancer must be ruled out.
– Endocervical or müllerian (10–15%): these are bilateral in at least 40% of cases and 20–30% are associated with ipsilateral endometriomas or pelvic endometriosis, as well as with BOT of mixed histology (seromucinous) [6].

## Diagnosis

Although the diagnosis of suspected BOT will be carried out using analytics, ultrasound, magnetic resonance imaging, and positron emission tomography (PET), as well as macroscopically, it is not possible to differentiate BOT from other ovarian tumours. The definitive diagnosis is histological. The histological criteria for diagnosis are: epithelial cell proliferation, stratified epithelium, microscopic papillary projections, cellular pleomorphism, nuclear atypia, and mitotic activity. In addition, there can be no stromal invasion, which is what differentiates them from invasive carcinomas [3, 6, 10].

However, in 10% of BOT, there are areas of microinvasion, with cells with the same features as BOT, defined by foci of < 5 mm or that do not invade the stroma > 10 mm2 [3, 11]. The stromal microinvasion is a controversial independent prognostic factor since it appears more frequently in serous BOT, and is associated with a higher frequency of micropapillary pattern and the appearance of peritoneal implants. It is considered a predictor of recurrence in invasive form [2, 7].

The peritoneal extension of BOT, called implants, are characterised as non-invasive (85%) when the epithelial proliferation affects only the peritoneal surface; while with invasive implants, there occurs in addition an extension to the underlying tissue, such as the omentum or intestinal wall [2, 6].

Once the BOTs are completely removed surgically they may recur, and may be of the borderline type (the majority), in which case survival is not affected, or of the invasive carcinoma type [2, 12], in which case, the prognosis of these patients may be drastically affected.

## Management and prognostic factors

The surgical treatment of BOT depends on the age of the patient, their reproductive wishes, the stage at diagnosis, and the presence or absence of invasive implants.

The FIGO stage classification is considered to be the greatest prognostic factor for recurrence and survival of BOT [1, 7], as it is in invasive carcinomas, but in contrast to these, the overall survival rate is greater. Published studies concluded that there was a 97–99% survival rate at five years when diagnosed at stage I, which decreased to 70–95% at ten years due to late recurrences [2, 3]; and to 65–87% in stages II and III at five years [3].

Surgical staging is based on operative findings, and consists in carrying out all procedures in the standardised clinical guides explained below [13], either in a first surgery or a second, if required, although there is a great deal of controversy around a second because it does not appear to affect patient survival [10]. A surgery will be considered ‘incomplete’ in cases where not all procedures were carried out, except in cases where preserving fertility was a concern, in which case, all procedures except hysterectomy and unilateral adnexectomy were performed [14].

Non-optimal staging in patients with BOT has a poor prognosis (Table 1), because without a deep peritoneal exploration, there could be invasive peritoneal implants. The importance of correct surgical staging lies in the need for a change in surgical treatment and postoperative adjuvant treatment if any added pathology is present. Theoretically, long-term survival would be diminished in patients with non-optimal staging with invasive implants, although the data do not seem to be statistically significant in the literature, probably due to the good overall prognosis of BOT and the low number of cases of each series [4, 7, 10, 14, 15]. In addition, non-optimal staging is considered a predictor of relapse, since women with incomplete surgery present a higher relapse rate, as high as double [7].

In spite of the fact that only 15% of unilateral tumours are associated with peritoneal extension, compared with 56% for bilateral, and with both radical and conservative surgeries as objectives, it would seem the most sensible course would be to perform complete surgical staging. However, this continues to be a topic for discussion. This surgery would be performed as an initial surgery upon getting an intraoperative diagnosis of BOT, or in a second surgery if the diagnosis was delayed after a chance intraoperative discovery, for example. It should be borne in mind that intraoperative analysis using fresh frozen samples tends to under diagnose BOT as benign tumours in 25–30% of cases, and carcinomas as BOT in 20–30% [2, 3, 7, 16].

### 1. Radical surgery

In postmenopausal women, and in those who have fulfilled their reproductive wishes, the following standardised procedures will be carried out: a thorough exploration of the abdominal cavity, bilateral salpingo-oophorectomy, total hysterectomy, inframesocolic omentectomy, peritoneal lavage to obtain samples for cytology, resection of macroscopically suspicious lesions, and multiple peritoneal biopsies (including omentum, intestinal serosa, mesentery, pelvic, and abdominal peritoneum), although this practice is in disuse due to its low sensitivity and the apparent lack of utility of randomised biopsies where no suspicious lesions are present [1, 3, 6].

In addition, in cases of mucinous BOT, appendectomies are performed to exclude ovarian metastasis whose origin is a primary carcinoma of the appendix.

Pelvic and paraaortic lymphadenectomy is not considered necessary since the involvement of lymph nodes does not decrease survival, and resection of these does not increase it. Lymphatic involvement, despite having no prognostic value in BOT, is an area associated with a recurrence or a progression to carcinoma, but this is exceptional and therefore justified by the morbidity associated with systematic lymphadenectomy in staging [3, 16].

It must be borne in mind that for women younger than 40, the diagnosis has a more favourable prognosis with a relative survival rate of 99% at five years. Nevertheless, the diagnosis worsens upon reaching the age of 70, when the five-year survival rate drops to 85%, probably in relation to the greater comorbidity related to the surgery and the postoperative period [1].

### 2. Conservative surgery

For women under the age of 40 who have not completed childbearing, a conservative treatment approach may be used if the patients are in stage I (with no peritoneal implants) [1, 3]; however, they should be informed that this treatment may decrease their fertility (prior rate of infertility is from 10–35%) due to the loss of ovarian tissue and pelvic adhesions [1–3]. The worst prognostic factor for recurrence is incomplete surgery, with recurrence rates of 10–20% as opposed to 5% after radical surgery, though these figures depend on the technique employed [3, 7].

In these cases, oophorectomy, unilateral salpingo-oophorectomy or cystectomy may be used, accompanied, just as with radical surgery, by the exploration of the cavity, omentectomy, peritoneal washing, resection of suspicious lesions, multiple peritoneal biopsies, and adnexectomy in mucinous BOTs. Routine biopsy on the contralateral ovary is not considered necessary unless an abnormality appears macroscopically, since it increases the risk of postoperative adhesions and yet is not of great value diagnostically, since it might not produce a tumour sample, as also occurs with multiple peritoneal biopsies [1, 3, 16].

With respect to adnexectomy, it should be borne in mind that this procedure appears to increase the risk of contralateral relapse [16]. In addition, cystectomy, which produces an increased risk of recurrence on the ipsilateral ovary (31%) [9], should be carried out only on women with bilateral tumours, with only one ovary, or on those patients who are extremely young, such that a loss of a large mass of ovarian tissue might negatively affect their fertility later on (though recent studies have obtained excellent fertility results in patients treated with unilateral salpingo-oophorectomy) [3, 7, 12, 17]. The increased relapse rate after cystectomy may be caused by: intraoperative cyst rupture, the presence of a multifocal BOT, or tumour margins affected after the cystectomy [1, 8]. Most of these recurrences are borderline type, so they do not affect global survival rates [3, 4, 7, 12, 17].

There has been much discussion as to whether conservative surgery, specifically, cystectomy, performed with laparoscopy could lead to higher relapse rates as compared with laparotomy, because of the increased risk of cyst rupture (14.9% versus 7.7%), incomplete staging, cellular dissemination, and increased trocar scarring [1–3]. In spite of this, most of the studies were carried out retrospectively, so that if the laparoscopy is performed by a trained specialist, it provides such benefits as lower morbidity and fewer postsurgical adhesions, as well as less pain and a shorter hospital stay [15].

In mucinous BOTs, cystectomy is not recommended as a treatment to preserve fertility due to the high risk of recurrence in the form of carcinoma (according to some studies up to 13% at ten years, compared with 2% at ten years for serous BOTs if not associated with invasive implants [17]). In addition, the possibility of the co-existence of benign, borderline, and invasive cancer areas has been described in mucinous BOTs especially of the intestinal type [2, 17], which implies that they should be carefully examined, given their great volume in some cases, and the treatment of choice will be salpingo-oophorectomy. For these reasons, mucinous BOTs are globally associated with a higher mortality rate. If survival is analysed according to histologic type, the worst results are found among patients with mucinous BOTs, with a global survival rate at ten years of approximately 94% as opposed to 96% for serous BOTs [3].

For women under the age of 40 who desire to have children and present with a BOT in stages II and III (with peritoneal implants), the surgical technique will vary according to the invasiveness of the implants:

– Non-invasive implants are benign, so that conservative surgery may be safely used as long as total resectioning of the peritoneal implants is carried out.
– Invasive implants: the presence of invasive implants is considered the second most relevant factor for a bad prognosis, although the majority of these implants remain stable or disappear when the primary tumour is removed [1, 2, 6, 7]. For those patients with invasive implants, radical surgery with complete re-sectioning of the implants is preferable [1, 3, 16].

According to previous studies, women without invasive implants have a survival rate at 10 years of 95%, since the disease progresses in only 2% of cases. However, for patients with invasive implants, the survival rate at ten years falls to 60–70% and progression of the disease to invasive tumour occurs in 30% of cases [1, 11, 14]. The risk of relapse for serious BOTs also depends on the invasiveness of the implants, at 11% for non-invasive implants, and rising to 45% for invasive implants at 15 years. Recurrence with transformation into carcinoma may occur in up to 77% of cases, which leads to an elevated mortality rate [6].

The debate continues over the possibility of completing the surgery in patients first treated with conservative surgery, through resectioning of the ipsilateral ovarian remnant and of the contralateral ovary as soon as these patients fulfil their childbearing desires. Hysterectomy seems unnecessary for these women, since the appearance of recurrences of serous uterine tumours has not been observed [3]. This treatment will only be indicated for those patients with BOTs with a high risk of recurrence (invasive implants, microinvasion, micropapillary patterns, or intracystic carcinoma) [1]. It may be possible to wait for recurrence to occur and then carry out radical surgery, since these conditions do not affect survival, probably because the majority occur in the spared ovary and can be successfully operated on. However, there is also the possibility of performing the radical surgery sooner because of the psychological impact produced by waiting for the relapse to occur, even risking recurrence in the form of an invasive tumour [14]. 

### 3. Surgery after recurrence

There are two types of surgical treatment (Table 2) for the ipsilateral ovary [3]:

– Conservative: all of the following requirements should be met: women < 40 years of age who want to preserve their fertility, who are committed to exhaustive follow-ups, and who do not have invasive implants.
– Radical: for cases that present some of the following: patients > 40 years of age, their childbearing desires completed, would find it difficult to adhere to follow-up requirements, and invasive implants.

When an extra-ovarian borderline or invasive relapse occurs, cytoreductive surgery as with primary ovarian cancer should be carried out. The optimum performance of this surgery is an independent prognostic factor, and will determine the patient’s survival, with death occurring in 12% of patients who were correctly treated as opposed to 60% of those who received insufficient treatment [3].

### 4. Adjuvant treatment

It has not been demonstrated that adjuvant treatment (chemotherapy or radiation therapy) improves the survival rate for patients with BOTs [1]. Response to the usual cytotoxic agents is low, probably related to the slow proliferation of these tumours. Neither do they seem to respond to oestrogen inhibitors in spite of being positive oestrogen receptors in 90% of cases. For this reason, there are no current indications for the use of chemotherapy or hormone therapy even in advanced cases.

The only situation where the usefulness of chemotherapy has been demonstrated is after surgery for serous BOTs with invasive implants, for which cases the chemotherapy regimen used is the same as that for invasive carcinoma (consisting of a platinum-containing drug, such as cisplatin or carboplatin, and a mitotic inhibitor, such as paclitaxel or docetaxel) [2, 4, 14, 16].

It appears that mutations in the KRAS or BRAF genes may produce cystoadenomas noted as serous BOTs, which could later evolve into low-degree serous carcinoma. In addition, mutation in the KRAS gene may be implicated in the origin of mucinous tumours, with their corresponding progression to mucinous carcinoma [3]. These lines of study may serve in the development new therapeutic targets efficient for BOTs, since drugs and their use in this respect are yet to be fully developed [2, 3].

## Follow-ups

Twenty-five percent of recurrences were diagnosed after five years [14], though relapses may actually occur 15 years after surgery, so patients must be closely monitored for a long time. Three follow-ups per year are recommended for the first two years, then one follow-up every six months during the next three to five years, and thereafter annually. Close monitoring is advised for women who were treated with conservative surgery because of the high rate of relapse.

Follow-up visits should include clinical exploration, transvaginal ultrasound, and Ca125 levels, even though some authors have suggested adding Ca19.9 since it appears that some mucinous tumours do not mark Ca125 [1, 6]. The importance of blood markers is controversial especially at early stages, since in earlier publications, only 40% of women diagnosed with a stage I BOT had elevated levels of Ca125, but if we look at the figures for stages II–IV the percentage rises to 83% [2]. When a relapse is suspected, transvaginal ultrasound is the test of choice, and may be accompanied by a pelvic RM. If evolutive peritoneal or extra-peritoneal disease is suspected, patient testing may also include CT scan or PET [6, 12].

## Conclusions

Younger women are more likely to be diagnosed with a BOT than with ovarian carcinoma, and their prognosis and global survival rates are much higher in comparison.

Choosing the best treatment for these patients is a real challenge. For those women who have not completed their childbearing desires, it appears safe to carry out conservative surgery as long as they do not also have invasive implants, and they do agree to remain under very close monitoring to ensure early diagnosis and treatment for future recurrences. However, not all professionals agree as to whether or not treatment should be completed with radical surgery once childbearing is complete, since it seems reasonable that not doing so increases the risk of recurrence. On the other hand, for women who do not desire to bear children, the consensus is that radical surgery should be practiced from the start.

In our opinion, it is crucial to perform complete staging in order detect the evolution of lesions, and administer the most appropriate adjutant treatment if necessary; however, there is ongoing discussion about performing restaging surgery on those patients with incomplete staging that might lead to a lower survival rate and increase the rate of relapse.

At the present time, it has not been shown that co-adjuvant treatments improve survival rates, except in cases of serous BOTs with invasive implants, which call for treatment with chemotherapy.

## Conflicts of interest

The authors have no conflicts of interest to declare.

## Figures and Tables

**Table 1.** Factors for bad BOT prognosis.

FIGO stages (II-III-IV)	Mucinous BOT
Invasive implants	Papillary pattern
Incomplete surgery	Microinvasion
Conservative surgery	Intracystic carcinoma
Age >40 years	Extraovarian relapse

**Table 2.** Factors that suggest a higher rate of invasive recurrence.

Serous BOT with invasive implants
Serous BOT with stromal microinvasion
Serous BOT with micropapillary pattern
Mucinous BOT with intraepithelial cancer
Mucinous BOT after cystectomy
Peritoneal involvement after surgery
